# Glioblastoma multiforme: a rare manifestation of extensive liver and bone metastases

**DOI:** 10.2349/biij.4.1.e3

**Published:** 2008-01-01

**Authors:** MC Robert, ML Wastie

**Affiliations:** Department of Biomedical Imaging (Radiology), Faculty of Medicine, University of Malaya, Kuala Lumpur, Malaysia

**Keywords:** Glioblastoma multiforme, GBM, extracranial metastases, glioma

## Abstract

Glioblastoma multiforme (GBM) is the most aggressive form of primary brain tumours known collectively as gliomas. Gliomas are graded by their microscopic appearance. As a rule, their behaviour can be predicted from histology: Grade I (pilocytic astrocytomas) and Grade II (benign astrocytomas) tumours are of low grade and grow slowly over many years. Grade IV tumours (GBM) are the most aggressive and, unfortunately, also the most common in humans, growing rapidly, invading and altering brain function. These tumours arise from the supporting glial cells of the brain during childhood and in adulthood.

These growths do not spread throughout the body like other forms of cancer, but cause symptoms by invading the brain. Untreated GBMs are rapidly lethal. Most patients with GBM die of their disease in less than a year and none have long term survival.

Extracranial metastases from GBM are extremely rare, with a reported frequency of only 0.44% because of the absence of lymphatics in the brain and the difficulty of tumours to penetrate blood vessels. A case of glioblastoma multiforme with the rare features of extensive liver and bone metastases is presented in this paper.

## INTRODUCTION

Gliomas are malignant brain tumours of glial cells growing along white matter tracts. Glioblastoma multiforme (GBM) is the most aggressive form of these primary brain tumours, which usually arise *de novo* or may develop from lower grade gliomas after many years. Distinct genetic alterations in GBM cases have been identified but as a rule there is no familial history. The peak age of presentation is in late adult life. Reported series from the USA give an incidence of approximately 20,000 cases of GBM per year.

GBM is usually located in the cerebral hemispheres, with a predilection for the white matter of the centrum semiovale and corpus callosum. There is relative sparing of the basal ganglia and grey matter. Posterior fossa and brain stem gliomas are seen in a younger age group. Multifocal tumours occur in 2-5% [1]. Contrast-enhanced CT scans of the brain demonstrate a variable tumour pattern from diffuse homogenous enhancement to heterogenous and ring enhancing patterns. The tumour, which rarely calcifies, usually extends beyond the margins of the enhancement. MR imaging demonstrates ill-defined tumour of a predominantly low signal on T1WI and heterogenous high signal on T2WI with slight gadolinium enhancement and usually accompanied by extensive vasogenic oedema. Areas of haemorrhage and central tumour necrosis may also be seen. Additional imaging methods are PET scanning where an increase in glucose utilization rates is seen and MR spectroscopy where increased choline peaks are noted [2].

The diagnosis of GBM requires tissue obtained by surgical methods. Surgery may vary from stereotactic needle biopsy of deep tumours to open surgical excision of surface tumours using frameless stereotaxis. As the name implies, GBM is multilobulated in appearance on gross pathology, demonstrating quite extensive vasogenic edema. It is a deeply infiltrating tumour with areas of haemorrhage and necrosis. Histologically, it is highly cellular, often bizarrely pleomorphic, undifferentiated multipolar astrocytes with prominent vascular endothelial proliferation and no capsule is demonstrable [3].

## CASE REPORT

**Figure 1:**
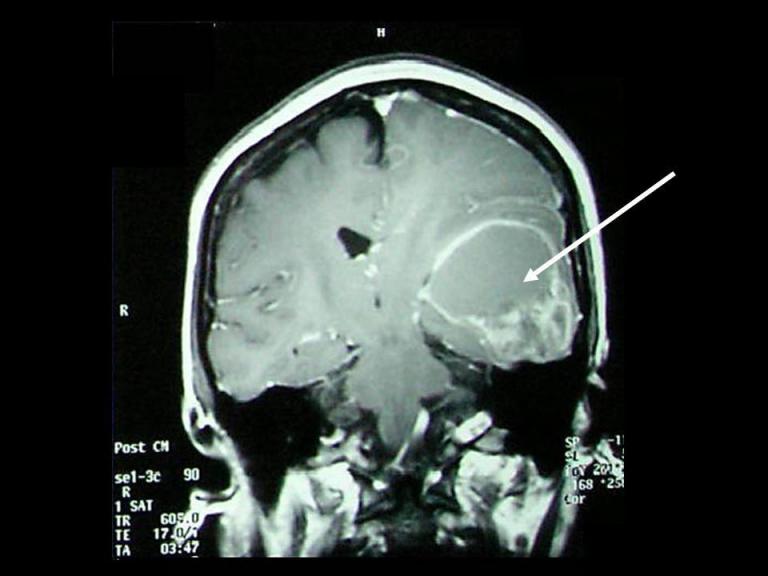
A coronal contrast-enhanced MRI of the brain demonstrating the heterogeneously enhancing left temporal lobe mass (glioblastoma multiforme) with an associated cystic component (arrow).

A 45-year-old woman had complained of progressive headache over a period of one year with associated disturbances in speech and blurring of vision over the last three weeks. A contrast-enhanced MRI of the brain was performed in February 2004, showing a heterogeneously enhancing lesion in the left temporal lobe with an associated cystic component (Figure 1). She then underwent a craniotomy with excision of the left temporal lobe tumour, which was diagnosed as a glioblastoma multiforme (WHO Grade 4) based on the histopathological findings. She received three cycles of radiotherapy, commencing three months post surgery. Post operatively, the patient’s condition improved and she was discharged well five days after the surgery.

**Figure 2:**
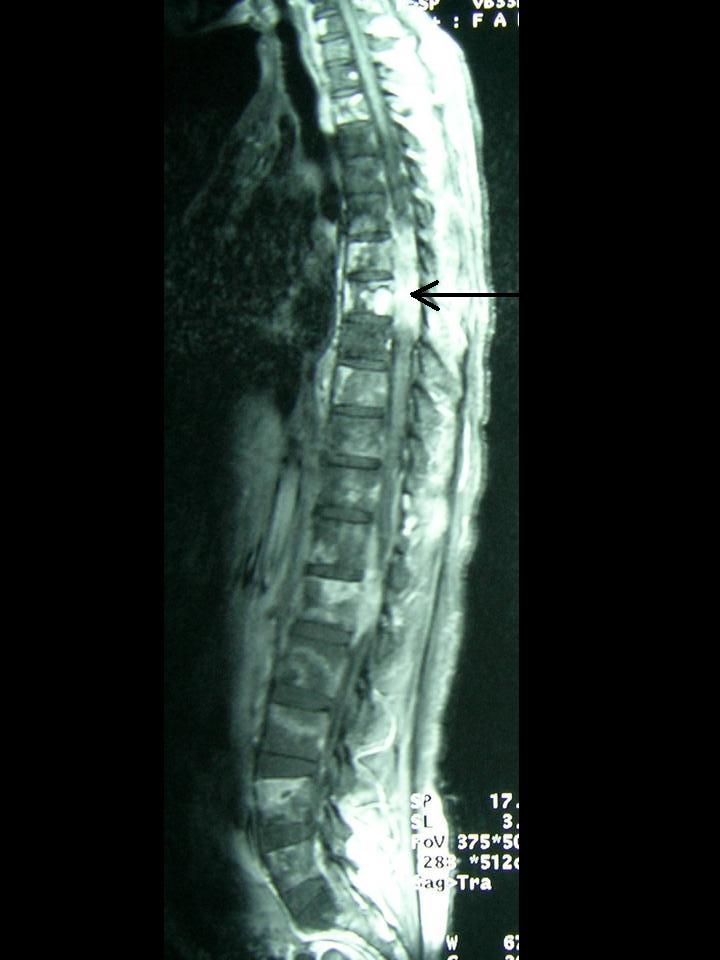
A contrast-enhanced T1 weighted sagittal MRI of the spine demonstrating multiple enhancing lesions in the vertebral bodies in keeping with bony metastases and cord compression at T7 level (arrow).

She then presented 9 months post-surgery with complaints of back pain. An MRI of the lumbosacral spine revealed features suggestive of bone metastases. A radionuclide bone scan performed two weeks later demonstrated extensive skeletal metastases. A subsequent MRI of the thoraco-lumbar spine demonstrated multiple areas of metastases with evidence of spinal cord compression at the T7 level (Figure 2).

**Figure 3:**
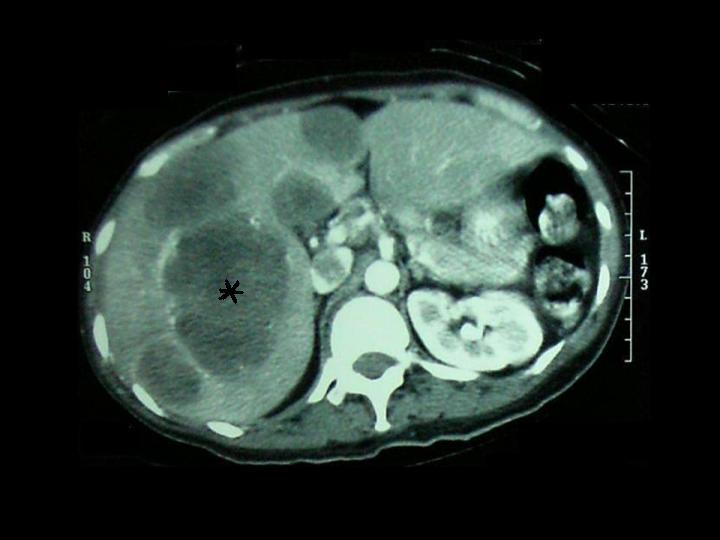
An axial contrast-enhanced CT scan of the liver demonstrating multiple liver metastases of varying sizes (the largest marked with an asterisk).

**Figure 4:**
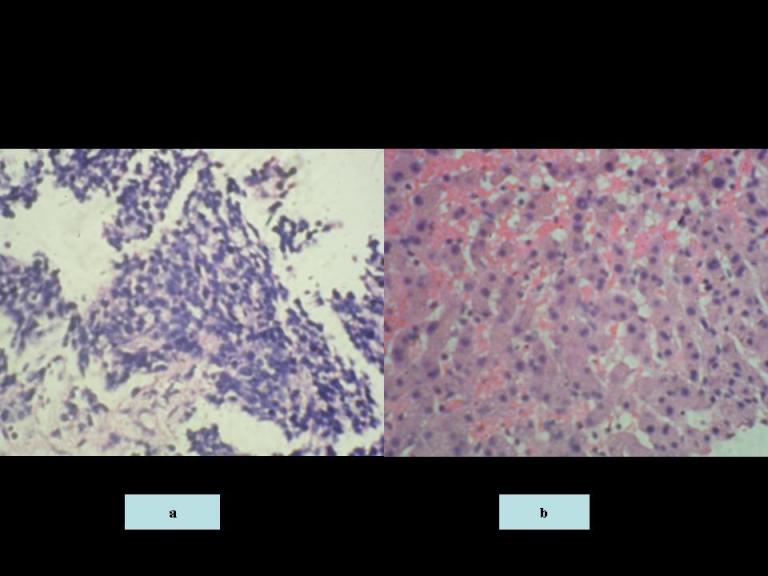
The H&E stained histopathological slide of the liver biopsy demonstrating a) abnormal sheets of small round ‘blue’ cells with high nucleocytoplasmic ratio and pleomorphism in keeping with glioblastoma multiforme metastases (slide magnification x200); b) the normal appearance of the hepatocytes obtained from a normal part of the liver tissue (slide magnification x500).

The patient by this time had also complained of vague abdominal pains, and a CT scan of the abdomen and pelvis showed multiple massive hypodense liver lesions of varying sizes (Figure 3). An ultrasound-guided core biopsy of one of these liver lesions was performed and was histopathologically confirmed to be that of liver tissue demonstrating abnormal sheets of small round ‘blue’ cells with high nucleocytoplasmic ratio and pleomorphism in keeping with glioblastome multiforme metastases (Figure 4a).

Based on the laboratory histopathology results and imaging findings, a diagnosis was made of glioblastoma multiforme (GBM) of the brain, with extensive liver and bone metastases.

## DISCUSSION

Extracranial metastasis of GBM is extremely rare, with a reported frequency of only 0.44%. Pasquier *et al*. found metastases occurred in regional lymph nodes (51%) and lungs and pleura (60%) and occasionally in the bone (31%) and liver (22%) [4]. Metastases from GBM are rare for a number of reasons: the cerebrum does not have a lymphatic system; the intra-cranial sinuses are enclosed in a dense dural membrane, which makes penetration by tumour cells difficult; intracerebral veins are thin walled and would probably collapse from compression before they could be penetrated by an expanding tumour; and the immunological response of the host organ to neuroglial tumour cells may prevent their growth outside the central nervous system [5]. A further explanation could be the short life span of patients with GBM who do not survive long enough to develop metastases.

Extra-cranial metastases have been described following stereotactic biopsy and few cases have been reported to occur in the absence of previous craniotomy [1]. Distant metastases generally occur after craniotomy, when direct access via the dural vessels to the extra-cerebral tissue is possible. When a tumour infiltrates the dura mater of the middle cranial fossa, metastases tend to occur in the lungs and pleura given the fact that much of the meningeal venous system flows back into the internal jugular venous system [2]. Haematogenous metastases may occur in the bone with the vertebrae being the most common site of bony involvement [6]. Lymph node metastasis is thought to occur through connections between perineural spaces and lymphatic plexuses [4].

In the present case, the patient was initially thought to have another malignancy giving metastases to the liver. However, histopathologic confirmation obtained via a liver biopsy revealed highly cellular pleomorphic cells similar to that seen in the primary cerebral glioblastoma multiforme. Therefore, the extensive liver lesions were accepted as extra-neural metastases of GBM and the patient was treated accordingly.

Much evidence now exists to support the notion that the removal of a maximum volume of tumour in the brain improves function and prolongs survival in the patients with GBM. Chemotherapy is a controversial treatment as many studies have failed to show prolonged median survival in treated patients, although the proportion of long term survivors may be somewhat greater. With distant metastases, chemotherapy is the only appropriate treatment but in these patients the prognosis is very poor.

## CONCLUSION

A case of glioblastoma multiforme of the brain with the rare occurrence of extensive extra-cranial metastases to the liver and bone is reported.

## References

[1] Anzil AP (1970). Glioblastoma multiforme with extracranial metastases in the absence of previous craniotomy. Case report.. J Neurosurg.

[2] Beauchesne P, Soler C, Mosnier JF (2000). Diffuse vertebral body metastasis from a glioblastoma multiforme: a technetium-99m Sestamibi single-photon emission computerized tomography study. J Neurosurg.

[3] He J, Olson JJ, James CD (1995). Lack of p16INK4 or retinoblastoma protein (pRb), or amplification-associated overexpression of cdk4 is observed in distinct subsets of malignant glial tumors and cell lines. Cancer Res.

[4] Pasquier B, Pasquier D, N'Golet A (1980). Extraneural metastases of astrocytomas and glioblastomas: clinicopathological study of two cases and review of literature. Cancer.

[5] Newton HB, Rosenblum MK, Walker RW (1992). Extraneural metastases of infratentorial glioblastoma multiforme to the peritoneal cavity. Cancer.

[6] Mihara F, Ikeda M, Rothman MI (1994). Vertebral body metastasis of glioblastoma multiforme with epidural mass formation. Contrast-enhanced MRI study. Clin Imaging.

